# Metabolic Changes in Peripheral Blood Mononuclear Cells Isolated From Patients With End Stage Renal Disease

**DOI:** 10.3389/fendo.2021.629239

**Published:** 2021-03-09

**Authors:** Mehmet M. Altintas, Salvatore DiBartolo, Lana Tadros, Beata Samelko, Haimanot Wasse

**Affiliations:** Department of Internal Medicine, Division of Nephrology, Rush University Medical Center, Chicago, IL, United States

**Keywords:** mitochondria, bioenergetics, PBMCs, ESKD, AMPK

## Abstract

As numerous complex pathologies stem from cellular energy dysfunction, we aimed to elucidate mitochondrial function and associated stress pathologies in kidney disease in a cohort of hemodialysis patients with end-stage kidney disease (ESKD). The bioenergetics study was conducted using peripheral blood mononuclear cells (PBMCs) of ESKD patients (n = 29) and healthy controls (no ESKD, n = 10). PBMCs were isolated from whole blood and seeded into assay plates to detect changes in oxidative phosphorylation and glycolysis. The bioenergetics analysis (i.e., mitochondrial stress test) was performed using Seahorse XFe24 flux analyzer. We observed significant reduction in mitochondrial respiration in patient PBMCs in terms of fundamental bioenergetics parameters such as basal respiration, ATP turnover, maximal respiration and spare respiratory capacity. These findings were correlated with the expression levels of proteins coordinating cellular energy status and regulating mitochondrial dynamics. Our data demonstrates an association between mitochondrial oxygen consumption of PBMCs and ESKD. AMPK activity, its downstream effector PGC-1α and mitochondrial fission/fusion proteins are partially responsible for the decrease in oxidative phosphorylation of PBMCs isolated from ESKD patients. We propose a link between mitochondrial dysfunction and ESKD and a role for mitochondria as a potential site for therapeutic interventions.

## Introduction

Chronic kidney disease (CKD) is a debilitating condition that can lead to progressive loss of kidney function and patients being placed on renal replacement therapy (1). It is defined as a decreased glomerular filtration rate (GFR) of less than 60 ml/min/1.73 m^2^ for at least 3 months and classified into five stages based on the GFR (2). CKD patients can progress to end stage kidney disease (ESKD) at stage 5, where GFR is less than 15 ml/min/1.73 m^2^.

Altered mitochondrial bioenergetics is central to the development and progression of a wide range of human diseases, including kidney diseases (3–5). Cells generate energy (in the form of ATP) necessary for cellular metabolism mainly through oxidative phosphorylation in mitochondria (6). Therefore, the measurement of mitochondrial function might be fundemantal to understanding the balance of various energy-intensive processes in kidney physiology.

Blood-based assays for mitochondrial function are definitive and minimally invasive tests used to reflect underlying disease conditions. We used PBMCs as the model system of disease monitoring for bioenergetic analyses. PBMCs are heterogeneous cell populations comprised of monocytes, T cells, B cells and natural killer cells and provide an accessible source of mitochondria and other sensitive biomarkers. Interestingly, PBMCs have capacity to mirror systemic changes within the body and PBMC bioenergetics have been investigated in several chronic diseases recently (7–10).

In this study, we used a population of ESKD patients and described a method allowing for measurement of mitochondrial oxygen consumption in PBMCs isolated from this population. We monitored mitochondrial respiration in PBMCs using Seahorse XF technology, which enabled us to report a quantitative analysis of oxidative phosphorylation and glycolysis by measuring oxygen consumption rate (OCR) and extracellular acidification rate (ECAR), respectively. It is well-established that this technique is capable of assessing mitochondrial function in terms of several bioenergetic parameters including basal respiration, ATP production, proton leak, maximal respiration, spare respiratory capacity and non-mitochondrial respiration in a highly sensitive manner (11, 12). Then, we related the PBMC bioenergetic parameters to alterations in other elements of mitochondrial biogenesis (AMPKα, p-AMPKα, Sirt3, PGC-1α), mitochondrial redox state and antioxidant responses (SOD2), and mitochondrial morphology (DRP1, OPA1), respectively. Our data support the notion that both oxidative and glycolytic metabolism are impaired in PBMCs isolated from ESKD patients, which might be a result of a contribution of various genetic and pathologic factors including the dysfunction of electron transport chain (ETC) machinery, limited availability of AMPK to restore intracellular energy levels and imbalance between mitochondrial fission and fusion processes compromising the ability of mitochondria to modify its morphology in response to environmental changes.

## Materials and Methods

### Study Design

A total of 50 subjects (35 ESKD in dialysis and 15 healthy subjects) were enrolled in our study. Since the extracellular flux analysis required a large number of cells, we did not include 11 blood samples (including 5 controls and 6 patients) with low PBMC yield. For protein expression analysis, we used the samples with sufficient amount of PBMCs left after the bioenergetics analysis (including all controls but only 11 patient PBMCs). Both cohort studies were carried out according to the principles expressed in the Declaration of Helsinki and the study protocol was approved by the Institutional Review Board of Rush University Medical Center. Written informed consent from each subject was obtained prior to inclusion.

### Isolation of PBMCs

Human PBMCs were isolated from freshly drawn blood (< 3 h after venipuncture) by standard density gradient centrifugation. Briefly, 7 to 10 ml of whole circulating blood from each donor were collected in a BD Vacutainer tubes (Becton Dickinson). The contents of the tube were diluted with an equal volume of Ca^2+^/Mg^2+^-free phosphate-buffered saline (PBS; Gibco) and layered over hyperosmolar Ficoll-Paque gradient (GE Healthcare) carefully without disturbing the interface. After centrifugation at 350*g* for 30 min at room temperature with the brakes off, the plasma at the top layer was removed. The mononuclear cells layered between plasma and Ficoll were collected with a disposable transfer pipet and transferred into a new 50-ml conical tube. The rest of the tube was filled with PBS and PBMCs were washed by spinning at 1,500 rpm for 10 min. The supernatant was removed carefully and the pellet containing PBMCs were suspended in PBS by gentle pipetting. The tube is filled with PBS again and the washing step is repeated one more time at similar conditions. After the last wash, the supernatant was pipetted off and the pellet was resuspended in 0.5–1.0 ml PBS using the 1-ml serological pipette. The PBMCs were then recovered with a final centrifugation (1,000*g* at 4°C for 5 min) and collected by the freezing medium containing RPMI-1640 medium supplemented with 30% FBS and 10% DMSO. An average of 5 to 10 million PBMCs were transferred into cryovials and stored at -80°C until the day of experiment.

### Preparing PBMCs for Bioenergetic Analysis

**Figure 1:**
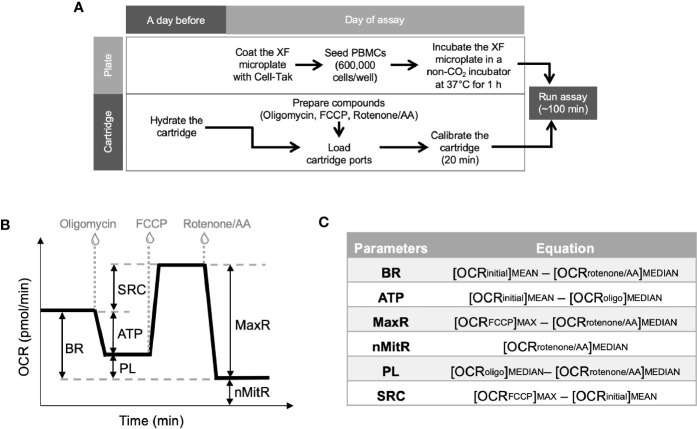
Experimental plan for extracellular flux analysis and the typical OCR curve with Mito Stress Test assay parameters. Cell culture plates were coated and PBMCs were loaded on the plates at the day of experiment whereas Seahorse XFe24 analyzer was stabilized and sensor cartridges were hydrated a day earlier **(A)**. Seahorse analyzer records OCR values (y-axis) versus time (x-axis) before and after the injections of oligomycin, FCCP and rotenone/antimycin A **(B)**. Using these OCR readings, the Mito Stress Test kit defines multiple key parameters of mitochondrial function including basal respiration (BR), ATP-linked respiration (ATP), spare (reserve) respiratory capacity (SRC), proton leak (PL), maximal respiration (MaxR) and non-mitochondrial respiration (nMitR) **(C)**.

On the day of analysis, vials were rapidly defrosted and the contents were placed in 10-ml prewarmed (37°C) culture medium (RPMI with 10% FBS, 100 U/ml penicillin and 100 μg/ml streptomycin). PBMCs were centrifuged at 200*g* for 5 min, the supernatant was removed. Cells were resuspended in fresh culture medium, counted and the viability was confirmed by trypan blue (Gibco) exclusion. PBMCs were then collected by spinning down at 200*g* for 5 min and washed once with the assay medium (Seahorse XF RPMI medium, pH 7.4, with 1 mM sodium pyruvate, 2 mM glutamine, and 10 mM glucose). By the meantime, we coated the Seahorse 24-well culture plate with 65 μg/ml Cell-Tak (Corning) tissue adhesive (in a solution containing 0.1 M sodium bicarbonate, pH 8.0 and 1 N NaOH) for 20 min. The wells were then washed twice with PBS to remove bicarbonate. After another centrifugation at 200*g* for 5 min, PBMCs were resuspended in the assay buffer and loaded into the wells of Seahorse 24-well culture plate along the side of each well. Specifically, we aimed to load 600,000 cells per well (in 150–200 μl) pertaining to our experience with PBMCs; however, the amount varies by the availability and the normalization was carried out based on the cell content. The plate was then centrifuged at 200*g* with no brake. The adhesion state and spreading of PBMCs on the coated well surfaces were visually confirmed under the phase-contrast microscope. Before bringing the plate to the analysis, the volume was brought up to 500 μl in each well by adding additional warm assay medium to each well slowly and gently. The plate was then transferred to a non-CO_2_ incubator set to 37°C for 45–60 min until loading it to the Seahorse analyzer (**Figure 1A**).

### Extracellular Flux Analysis

A day before the experiment, Seahorse XFe24 Analyzer (Agilent) was turned on with Wave software (Agilent) running overnight to stabilize the analyzer. At the same time, Seahorse XFe24 sensor cartridge (Agilent) was hydrated by filling each well of the 24-well utility plate with 1 ml of calibration solution (Agilent) and submerging sensors into it. Then, the stack of utility plate and the sensor cartridge (FluxPak) was placed in a non-CO_2_ incubator set to 37°C overnight. On the day of experiment, FluxPak was taken out of the non-CO_2_ incubator and the injection ports were filled with the following compounds: oligomycin (an inhibitor of oxidative phosphorylation), fluoro-carbonyl cyanide phenylhydrazone (FCCP, a mitochondrial uncoupler), and rotenone/antimycin A (inhibitors of complex I/complex III). These compounds were provided as lyophilized powders in Mito Stress Test Kit (Agilent), which was used to investigate mitochondrial function in this study. We first prepared the stock solutions by resuspending those in assay medium and then further diluted to 10X concentrations before loaded into the ports. We used 1.0 μM oligomycin and 0.5 μM of each of rotenone and antimycin A for each specimen. However, we had to titrate the FCCP concentrations (test-range of 1.00 to 2.00 μM, with 0.25 μM increments) and choose the optimal concentration yielding the maximal oxidative capacity for each sample, since the concentration of FCCP varies according to the metabolic demands of the cells (11). Besides, FCCP can be inhibitory at high concentrations (13). The loading volumes of each 10X compounds into the injection ports are 56 μl oligomycin, 62 μl FCCP, and 69 μl rotenone/antimycin A. When the calibration process was over (after about 20 min), the the utility plate was replaced with the culture plate. After a short (3 min) mixing period, the first set of measurements were recorded under basal conditions. After three recordings, oligomycin, FCCP and rotenone/antimycin A were injected successively into each well. Between each injection, three measurements of each of OCR and ECAR were recorded. All recordings were 8 min apart and the running time for a standard mitochondrial stress test was about 100 min.

### Calculation of Metabolic Parameters for Mitochondrial Function

Together with all the compounds and injection strategies, Mito Stress Test analysis provided measurements of basal respiration, ATP production, maximal respiration, spare respiratory capacity, non-mitochondrial respiration, and proton leak. **Figure 1B** demonstrates typical OCR curve and the mitochondrial stress assay parameters. Those metabolic parameters were calculated for each well using the OCR plot as explained in **Figure 1C** (12). Then, averages (and error bars) of individual well results (n = 4 or 5) for each sample (control or patient) were calculated. Since PBMCs were counted immediately before the assay and equal numbers of PBMCs were plated per well for each sample, Seahorse data were internally normalized to cell count.

Two additional factors (e.g., coupling efficiency and bioenergetic health index) were derived from those parameters to characterize the bioenergetics of PBMCs. Coupling efficiency of the ETC is expressed as the ratio (or percent) of the proton motive force dissipated by the ATP synthase (i.e., the ratio of ATP turnover to basal respiration). It is a measure of oxygen consumed to drive ATP synthesis compared to that driving proton leak. The other parameter, bioenergetic health index (BHI), incorporates various bioenergetic parameters and can be defined as (14):

(1) $$BHI=log\frac{{\left[Spare\;respiratory\;capacity\right]}^{a}\times {\left[ATP\;production\right]}^{b}}{{\left[Non-mitochondrial\;respiration\right]}^{c}\times {\left[Proton\;leak\right]}^{d}}\;$$

No power function was applied (i.e., exponents a, b, c, and d were all set to 1.0). It acts as a surrogate index of changing bioenergetic health of individuals.

Another measurement of metabolic activity is extracellular acidification and recorded as ECAR. The major contributor of medium acidification is lactate production in the extracellular medium. Assuming that there was a reliable correlation between ECAR and lactate production, changes in basal ECAR in adherent PBMCs was used to estimate the end-point lactate concentration by the cells, which reflected their glycolytic capacity. We calculated basal rates of medium acidification (OCR/ECAR at basal respiration) to predict the glycolytic reserve capacities of PBMCs at resting conditions. Furthermore, the acute increases in medium acidification in response to oligomycin (OCR/ECAR after adding oligomycin) and acute increases in medium acidification in response to FCCP (OCR/ECAR at maximal respiration) were also calculated to estimate glycolytic capacities when mitochondrial ATP production was blocked by oligomycin, and when artificial ATP demand was induced by FCCP, respectively.

### Western Blot Analysis

For whole cell lysates, PBMCs were resuspended in ice-cold lysis buffer containing 50 mM Tris-HCl, pH 7.4, 150 mM NaCl, 1% Nonidet P-40, 0.5% sodium deoxycholate, 0.1% sodium dodecyl sulfate (SDS), 5 mM EDTA, 1 mM EGTA (Boston BioProducts) supplemented with protease (Roche Diagnostics) and phosphatase inhibitors (Sigma Aldrich). After incubation on ice for 30 min (including five pulse vortexing steps), lysates were centrifuged at 13,000 rpm for 15 min at 4°C to discard the cell debris. The lysates were adjusted for protein content using Bradford assay (Bio-Rad) and mixed with LDS sample loading buffer and reducing agent (both from Thermo Fisher Scientific) before being denatured by heating at 70°C for 10 min. Proteins were separated by electrophoresis using NuPAGE Novex 4–12% Bis-Tris protein gel (Thermo Fisher Scientific) and transferred to Immobilon-P PVDF membrane (Millipore) according to the manufacturer’s instructions. The following primary antibodies were used for immunoblotting: AMPKα (1:1,000; Cell Signaling Technology, 2532), p-AMPKα (Thr172) (1:1,000; Cell Signaling Technology, 2535), β-actin (1:1,000; Cell Signaling Technology, 3700), CoxIV (1:1,000; Cell Signaling Technology, 4844), DRP1 (1:1,000; Cell Signaling Technology, 14647), lactate dehydrogenase (1:1000; Cell Signaling Technology, 2012), OPA1 (1:1000; Cell Signaling Technology, 80471), PGC-1α (1:1000; Cell Signaling Technology, 2178), PDHK1 (1:1000; Cell Signaling Technology, 3820), Sirt3 (1:1000; Cell Signaling Technology, 2627), and SOD2 (1:1000; Cell Signaling Technology, 13141). Following the overnight incubation with primary antibodies at 4°C, membranes were probed with either anti-rabbit (Promega, W401B) or anti-mouse (Promega, W402B) HRP-conjugated secondary antibodies (both 1:10,000) for 1 h. Proteins were detected with ECL Reagent (Thermo Fisher Scientific) and autoradiography films (Denville) were developed using an X-ray film processor (Alphatek). Signal intensities were measured by densitometry using Image J software (NIH).

### Statistical Analysis

Experiments for mitochondrial function analysis were replicated in four or five wells whereas the protein expression analyses were independently repeated two or three times. Results were averaged for each treatment group. Data are presented as means ± standard errors of the means (SEM). Student’s t-test was used to assess differences in experimental features by using Prism 6 software (GraphPad). Significant differences compared to controls (CTL) are annotated as follows: *P < 0.05, **P < 0.01, and ***P < 0.001.

## Results

### Study and Participant Characteristics

A total of 29 patient samples and 10 controls had sufficient PBMCs for metabolic analysis (**Table 1**). Ninety percent of patients had hypertension and 62% had diabetes.

**Table 1:** The main demographic and clinical features of study group (HTN, Hypertension; DM, Diabetes mellitus; F, Female; M, Male; AA, African American; CAU, Caucasian; HSP, Hispanic).

	Healthy Subjects	ESKD Patients
Number	10	29
Disease status (HTN/HTN and DM/Others)	–	(8/18/3)
Age (Range/Median)	(25–64/40)	(35–80/61)
Gender (F/M)	(7/3)	(17/12)
Race (AA/CAU/HSP)	(3/6/1)	(22/3/4)

### Oxygen Consumption Rates and Bioenergetic Parameters of Controls and ESKD patients

**Figure 2:**
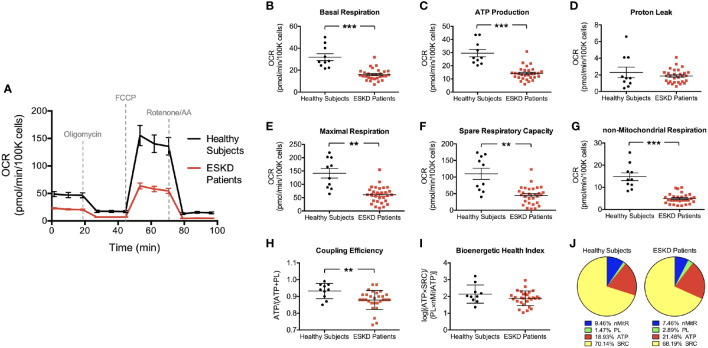
Mitochondrial profiles of PBMCs isolated from ESKD patients and healthy controls. Oxygen consumption profiles of patient PBMCs (disease, n = 29) and healthy controls (health, n = 10) recorded before and after adding oligomycin, FCCP, and rotenone/antimycin A, respectively **(A)**. The assay reports significantly higher basal respiration **(B)**, ATP-linked respiration **(C)**, maximal respiration **(E)**, spare respiratory capacity **(F)** and non-mitochondrial respiration **(G)** in PBMCs of healthy subjects compared to those in PBMCs from ESKD patients. Both groups have similar levels of proton leak **(D)**. Coupling efficiency **(H)** and bioenergetic health index **(I)** were also assessed using these parameters. Pie charts show the percentile contribution of each of ATP-linked respiration (ATP), proton leak (PL), spare respiratory capacity (SRC) and non-mitochondrial respiration (nMitR) when maximal OCR is established as 100% in health and disease **(J)**. **P < 0.01 and ***P < 0.001.

In order to examine oxygen consumption rates and bioenergetic parameters of controls and ESKD patients, we used the Seahorse XFe24 analyzer in a live cellular environment (**Figure 2A**).

We first established the “resting” OCR (OCR_initial_), which accounts for the basal rate of respiration in PBMCs after removing the contribution from non-mitochondrial oxygen consumption (**Figure 1B**). We found that PBMCs isolated from healthy individuals had a significantly higher basal respiration when compared to that from ESKD patients (31.9 ± 3.1 vs. 16.0 ± 1.0 pmol/min/10^5^ cells, P < 0.001) indicating a much higher endogenous ATP demand in control cells (**Figure 2B**).

Then, PBMCs were treated with oligomycin, which decreases electron flow through ETC by inhibiting ATP synthase. The resulting reduction in mitochondrial respiration after oligomycin injection (OCR_oligo_) isolates the OCR linked to ATP production (**Figure 1B**). Oxygen consumption that is used to produce mitochondrial ATP was higher in control PBMCs than those isolated from patients (29.6 ± 2.8 vs. 14.1 ± 0.9 pmol/min/10^5^ cells, P < 0.001, **Figure 2C**) reflecting a reduced ability of patient PBMCs to use membrane potential, which serves a driving force for ATP synthesis (15). We also measured the energy dissipation as proton leak (**Figure 1B**) and found that both groups demonstrated similar proton leak-linked respiration (2.3 ± 0.6 and 1.9 ± 0.2 pmol/min/10^5^ cells in health and disease, respectively) as shown in **Figure 2D**.

Subsequent treatment with a mitochondrial uncoupler, FCCP, shuttles protons across the inner membrane causing ETC to operate at maximal capacity (16). The resulting effect was the maximum OCR reading (OCR_FCCP_) maintaining the proton motive force at the highest levels (**Figure 1B**). PBMCs from healthy individuals was able to reach higher levels of oxidative respiration following FCCP treatment than those from patients (141.6 ± 17.9 vs. 60.8 ± 5.8 pmol/min/10^5^ cells, P < 0.01, **Figure 2E**). Obtaining the maximal respiration curve also allowed us to calculate the spare (or reserve) respiratory capacity, i.e., the difference between the FCCP-stimulated and resting OCR (**Figure 1B**). PBMCs obtained from healthy controls had substantially higher reserve respiratory capacity than that from patients (109.7 ± 16.7 vs. 44.8 ± 5.0 pmol/min/10^5^ cells, P < 0.01, **Figure 2F**).

As a final step of the mitochondrial stress test, rotenone and antimycin A were added to block complexes I and III, respectively. Together, these agents fully inhibit the mitochondrial electron transfer and the residual OCR measured can be attributed to non-mitochondrial oxidases (OCR_rotenone/AA_). This residual respiration was almost three times higher in PBMCs isolated from controls than those from patients (14.8 ± 16.7 vs. 4.9 ± 5.0 pmol/min/10^5^ cells, P < 0.001, **Figure 2G**) indicating that non-mitochondrial respiration contributes to the overall respiration to a greater extent in control PBMCs than patient PBMCs.

We found that the coupling efficiency is lower in PBMCs from ESKD patients (0.93 ± 0.01 vs. 0.88 ± 0.01, P < 0.01, **Figure 2H**) suggesting that the mitochondrial efficiency of substrate oxidation is less in those cells compared to healthy controls. On the other hand, BHI was not significantly different in both groups (2.1 ± 0.2 vs. 1.9 ± 0.1, **Figure 2I**). This finding is surprising since we were expecting that mitochondrial health was impaired in patient PBMCs considering the relatively low performance of those cells in terms of various bioenergetic parameters (**Figures 2B, C, E–H**). This can be explained by the fact that each parameter in this equation contributes similarly to the overall respiration in both groups, i.e., proton leak supplies 1.5% (controls) to 2.9% (patients), non-mitochondrial respiration supplies 7.5% (patients) to 9.5% (controls), ATP production supplies 18.9% (controls) to 21.5% (patients) and spare respiratory capacity supplies 68.2% (patients) to 70.1% (controls) of the overall respiratory capacity services (**Figure 2J**). Otherwise, our BHI assessment (Equation 1) needs to be modified with updated exponents to predict the health status in a comprehensive manner.

### Extracellular Acidification by PBMCs

**Figure 3:**
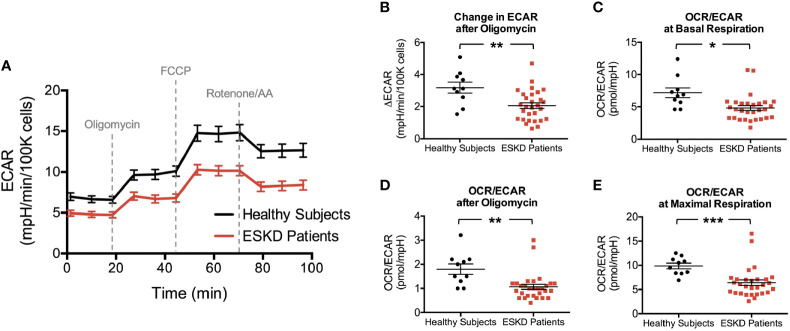
Rates of extracellular acidification in PBMCs due to lactate and CO_2_ production through glycolysis and mitochondrial respiration, respectively. Extracellular acidification is measured to be higher in PBMCs of healthy subjects throughout the analysis **(A)** yielding a stronger response after oligomycin in this group **(B)**. The corresponding OCR : ECAR ratios in PBMCs at basal respiration **(C)**, following oligomycin treatment **(D)** and at maximal respiration **(E)** were also reported to be higher in healthy subjects than those of ESKD patients. *P < 0.05, **P < 0.01, and ***P < 0.001.

We were also able to detect the glycolytic function of PBMCs by measuring the extracellular acidification rates (ECAR) in our cells, which indicate the levels of lactic acid generated by glycolysis (**Figure 3A**). ECAR curve was similar to OCR curve except the addition of oligomycin caused loss of OCR (**Figure 2A**) but a compensatory increase in ECAR (**Figure 3A**), i.e., glycolysis is upregulated in response to the inhibition of mitochondrial oxygen consumption. We first evaluated the change in extracellular acidification (ΔECAR) in response to oligomycin (**Figure 3B**) using the overall ECAR plot (**Figure 3A**). PBMCs from healthy subjects had more robust response to oligomycin (3.2 ± 0.3 vs. 2.1 ± 0.2, P < 0.01) indicating that, compared to PBMCs from ESKD patients, these cells more readily switched to glycolysis when ATP synthase (and mitochondrial respiration) was inhibited.

Then, we analyzed OCR/ECAR ratios to assess the relative contribution of glycolysis and oxidative phosphorylation to energy generation (**Figures 3C–E**). We found higher OCR/ECAR ratios at the baseline (7.2 ± 0.8 vs. 4.8 ± 0.4, P < 0.05, **Figure 3C**), following the blockade of ATP synthase (1.8 ± 0.2 vs. 1.1 ± 0.1, P < 0.01, **Figure 3D**), and maximal respiration conditions (9.9 ± 0.6 vs. 6.4 ± 0.6, P < 0.001, **Figure 3E**), consistent with bioenergetic profiles of both groups (**Figures 2A** and **3A**).

### Integration of Metabolic Analysis With Western Blotting

**Figure 4:**
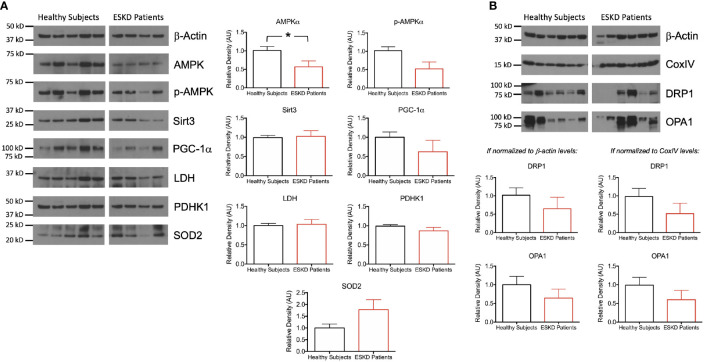
Functional units of cell metabolism regulating bioenergetic machinery. **(A)** Representative Western blots of PBMC lysates (left) from ESKD patients (n = 11) and healthy controls (n = 10) for key proteins, which are involved in bioenergetics and participate in mitochondrial function (AMPKα, p-AMPKα, Sirt3, PGC-1α, LDH, PDHK1 and SOD2) and corresponding densitograms (right). **(B)** Representative Western blots of mitochondrial fission (DRP1) and fusion (OPA1) proteins as well as the loading control antibodies (top) and their band intensities relative to the house keeping proteins β-actin (bottom, left) and CoxIV (bottom, right). *P < 0.05.

We measured the expression levels of a subset of mitochondrial proteins and others, which influence the energy metabolism of cells (**Figure 4**). AMP-activated protein kinase (AMPK) is one of these key regulators of energy homeostasis (17, 18). We found that AMPKα expression and activation (i.e., phosphorylation of AMPKα on Thr172) were significantly higher in control PBMCs compared to patient PBMCs (P < 0.05 for both, **Figure 4A**). Sirtuins represent another important group of energy sensor (19) and sirtuin 3 (Sirt3) is an emerging regulator of mitochondrial bioenergetics and ATP generation (20). Sirt3 activation was shown to increase mitochondrial respiration (21, 22) but we found comparable levels of expression of this mitochondrial matrix protein in both groups (**Figure 4A**). Next, we tested peroxisome proliferator-activated receptorγ (PPARγ) coactivator-1α (PGC-1α) levels as it is an essential transcription factor regulating mitochondrial oxidative metabolism and energy expenditure (23, 24). Western blot analysis against PGC-1α antibody revealed a higher expression in PBMCs from healthy subjects (**Figure 4A**).

We then focused on the pyruvate node, which connects glycolysis to tricarboxylic acid (TCA) cycle. We sought the substrate catabolic pathways feeding the TCA cycle to see whether the low respiration rates in PBMCs from ESKD patients were due to i) direction of glycolysis products to lactate rather than TCA cycle or ii) blockade of the catalysis of pyruvate to acetyl-CoA, which enters the TCA cycle for subsequent oxygen-dependent energy production processes. The former is catalyzed by lactate dehydrogenase (LDH) (25) whereas the latter was catalyzed by pyruvate dehydrogenase (PDH) but suppressed by pyruvate dehydrogenase kinases (PDHKs) (26). We found comparable levels of each of LDH and PDHK1 in both groups (**Figure 4A**), suggesting that the dramatic differences in oxidative phosphorylation between control and patient groups were not due to the control over this metabolic switch around pyruvate.

Next, we wanted to investigate the oxidative stress-related mechanisms by assessing the antioxidant status of our PBMCs. We analyzed the expression of the mitochondrial-specific free radical scavenging enzyme, superoxide dismutase 2 (SOD2) and found a higher expression in PBMCs from ESKD patients (**Figure 4A**) indicating a rise in the antioxidant defense system, which has probably been correlated with the higher levels of oxidative damage in those cells (27).

In order to investigate the link between mitochondrial function and morphology, we interrogated the levels of DRP1 and OPA1, which are involved in mitochondrial fission and fusion, respectively. Both mitochondria-shaping proteins were expressed less in patient PBMCs than that in healthy subjects when the expression levels were normalized for the internal reference antibodies β-actin (cytoskeleton) or CoxIV (mitochondrial) (**Figure 4B**) implicating a dysfunction in mitochondrial dynamics.

## Discussion

In this study, we determined the oxygen consumption rates in PBMCs isolated from ESKD patients, which are associated to mitochondrial respiration. We found that basal, ATP-linked, and maximal oxygen consumption in the patient group were lower than those in control PBMCs, indicating an impaired mitochondrial function that lead to a reduction of the components of ETC machinery and a decrease in oxidative phosphorylation in kidney disease. Even though oxidative phosphorylation is tightly coupled to electron transport under normal conditions, the energy produced during substrate oxidation is not entirely coupled to ATP synthesis. Some protons, which are pumped from the mitochondrial matrix to the mitochondrial intermembrane space to enter the ETC, leak back across the mitochondrial inner membrane *via* uncoupling proteins (28). The level proton leak was comparable in health and disease undermining its impact on the mitochondrial performance. Another important parameter was reserve respiratory capacity, which is indicative of how well the cells are equipped for increasing their capacity of mitochondrial ATP synthesis (29) and measured by change in oxidative phosphorylation after uncoupler stimulation. Control PBMCs have a better ability to maintain a reserve of energy generating capacity and respond to an unexpected increase in energy demand (e.g., when there is an acute insult such as drastic changes in ion pump activation). PBMCs isolated from the ESKD patients could not reach as high levels of oxidative phosphorylation achieved by control PBMCs following uncoupler stimulation, which might be due to the reduced mass or compromised integrity of mitochondria (30). Consistently, coupling efficiency was lower in patient PBMCs mainly due to respiration associated with alternative proton conductance pathways in those cells, which generally results in heat production at the expense of ATP synthesis (31).

We also measured extracellular acidification rates, which are ascribed to glycolysis. Actually, various pathways other than glycolysis cause extracellular acidification such as bicarbonate (32) and respiration-derived carbon dioxide (CO_2_) (33). To eliminate its contribution to acidification, bicarbonate was eliminated from the assay medium; otherwise, it would bind to protons generated from glycolysis and dissociate to form water (H_2_O) and CO_2_, which would eventually degas and cause changes in pH. It was more challenging to correlate the contribution of CO_2_ since it required to perform the mitochondria stress test analysis using a glucose-free assay medium. In that case, acidification would be mostly due to CO_2_ hydration and dissociation rather than glycolysis, which would exhibit an ECAR pattern representing CO_2_ contribution alone. Due to the limited availability of control and patient samples, we did not follow this strategy to subtract the contribution of CO_2_ produced during mitochondrial activity (i.e., energy generation in TCA cycle) but assumed that lactate concentration by PBMCs reflected their glycolytic capacity (34). Therefore, we might end up with slightly increased ECAR values as CO_2_ contributes substantially to extracellular acidification. The assay produced ECAR curves similar to OCR plots, i.e., glycolysis was upregulated in a greater extent in control cells compared to patients when mitochondrial oxygen consumption is inhibited. Our findings suggested that energy is predominantly generated by oxidative phosphorylation in PBMCs from healthy subjects and PBMCs from ESKD patients had relatively higher reliance on glycolysis when compared to control PBMCs. However, these results did not rule out neither the existence of a robust glycolytic activity in control PBMCs nor the residual level of oxidative phosphorylation in patient PBMCs.

Further analysis of the pathways feeding the TCA cycle at the pyruvate node (i.e., the final stages of glycolysis) yielded that the protein expression levels of each of LDH, which reduces pyruvate to lactate, and PDHK1, which suppresses pyruvate dehydrogenase complex and inhibits the conversion of pyruvate to acetyl CoA were similar in healthy subjects and ESKD patients. The former mechanism allows a subsequent excretion of lactate to the extracellular space, whereas acetyl CoA conveys the carbon sources to TCA cycle for energy production (so, the activities of both enzymes serves to redirect pyruvate to lactate production rather than TCA cycle). These findings point out that the poor respiration in patient PBMCs might not be due to the enzymes or metabolites associated with the pyruvate node but the production of pyruvate itself since the analysis of the glycolytic function in patient PBMCs suggested that this pathway was also performing at a lower rate than the controls. One little note here is that even though the rate of glycolysis and pyruvate production was estimated by means of the extracellular acidification rate [ECAR (34)], there are reactions within the glycolysis pathway that contribute to acidification and are not associated with lactate production (35).

One pathway that stands out as significant in patient PBMCs is the AMPK pathway. AMPK is a serine/threonine kinase and is known to mediate several signaling pathways to maintain mitochondrial function. It is activated when AMP : ATP ratio is increased as a cellular response to stress, and once this happen it exerts a pivotal role in compensating reduced ATP levels (36). In other words, it senses the ATP depletion and then turns on ATP-producing reactions while switching off ATP-consuming reactions. It regulates various metabolic processes and is directly involved or dysregulated in major chronic diseases (37, 38). Both AMPK and its activated form (through phosphorylation at a threonine residue, Thr-172, of the α-subunit) were expressed at significantly lower levels in PBMCs from ESKD patients, which highlights its essential role in maintaining cellular energy homeostasis in these patients. In healthy subjects, we also found high levels of PGC-1α, which is reasonable since the rate of oxidative phosphorylation was measured to be higher in control PBMCs compared to patient PBMCs and high PGC-1α levels were implicated with increasing bioenergetic demands (39). Interestingly, animal studies demonstrated that fibroblasts derived from PGC-1α null mice had a greater sensitivity to oxidative stress compared to wild type fibroblasts (40), which might explain the significantly compromised mitochondrial respiration in patient PBMCs. This finding also reflected the results of AMPKα blots since AMPK was reported to activate PGC-1α both *in vivo* and *in vitro* (41). Moreover, mRNA levels of several PGC-1α downstream target genes were found to be profoundly down-regulated in PBMCs isolated from dialysis patients (42).

Emerging research evidence has suggested that disruption of mitochondrial respiration and ETC activity resulted in generation of free radicals, which eventually contributed to increased oxidative stress (43). In the next step, we wanted to see how PBMCs respond to oxidative stress by evaluating the expression levels of an essential mitochondrial antioxidant enzyme SOD2, which catalyzes the conversion of superoxide to hydrogen peroxide and molecular oxygen by binding to the superoxide byproducts of oxidative phosphorylation (44, 45). We found a higher expression of SOD2 in patient PBMCs, which is in agreement with earlier studies reporting higher SOD2 gene expression in hemodialysis (46) and peritoneal dialysis patients (42) compared to healthy controls. Other studies also reported increased mitochondrial reactive oxygen species (ROS) in CKD (47) or ESKD patients (48) and upregulation of anti-oxidative regulatory genes in PBMCs of either CKD or ESKD patients (49). In support of these findings, AMPKα-deficient mouse embryonic fibroblasts exhibited 50% higher mitochondrial ROS implicating that AMPK acted as a sensor of mitochondrial ROS production and subsequent expression of antioxidant genes (50).

Mitochondria are highly dynamic organelles that constantly change their shape and number *via* the well-characterized mechanisms, fission and fusion, mainly to repair, adapt or meet energy demands (51). Mitochondrial dynamics was also implicated as an important process contributing to mitochondrial dysfunction (52, 53). Therefore, we wanted to see the expression levels of two major proteins of the mitochondrial morphogenesis machinery, DRP1 (54, 55) and OPA1 (56, 57), that are involved in mitochondrial fission and fusion, respectively. We found that both proteins were less abundant in PBMCs in ESKD patients compared to that in healthy subjects indicating a disequilibrium between relative rates of fission and fusion. This might alter mitochondrial ultrastructural morphology and potentially disrupt mitochondrial function in PBMCs isolated from ESKD patients. There is also evidence that AMPK can regulate the mitochondrial fission and fusion machinery to mediate these events (58) that might explain the reduced levels of DRP1 and OPA1 in ESKD cells displaying low AMPK activity.

A limitation of the current study is that our control subjects were younger than patients. Genetic differences might also occur since the racial composition varied across the population of each group (**Table 1**). These disparities with regard to age and race must be considered when interpreting our findings. Since PBMCs are heterogenous population of immune cells and each subpopulation might have a unique bioenergetic profile (59), the failure to determine the proportion of each cell type in healthy subjects and ESKD samples is a weakness of this study. On the other hand, PBMCs are likely to reflect the overall metabolic state and provide an ideal model for monitoring systemic changes (60). In recent years, the oxygen consumption characteristics of PBMCs are used as an index of metabolic function in a wide range of diseases such as type 2 DM (7), chronic fatigue syndrome (9), human immunodeficiency virus (HIV) infection (10), Alzheimer’s disease (61), and sepsis (62). In addition, the sample size was relatively modest. Moreover, we were not able to test all of the samples with an equivocal Western Blot analysis due to the limited amount of sample volume and the number of samples used for this analysis varied by the availability of the PBMCs in each of healthy and disease group. It would be interesting to validate these results in a larger population. Challenges of patient recruitment also affected our study design in a way that we collected blood samples and isolated PBMCs on the day of patient visit but had to freeze PBMCs for bioenergetic analysis later. Even though studies examining the mitochondrial function in blood cells generally use freshly isolated cells, cryopreservation can be used in the design of various studies including leucocytes (63), lymphocytes (12) and PBMCs (9). Quality assessment reports on cryopreservation are also available suggesting the frozen PBMCs -if properly cryopreserved- as an attractive alternative to freshly isolated cells for functional studies (64, 65). Indeed, these limitations are the subject of further improvements but can be balanced by the applicability and efficacy of our methodology.

Taken together, these experiments with PBMCs show that a mitochondrial impairment has been consistently reported for patients with ESKD and highly reduced bioenergetic parameters may be a feature of these patients. In addition, we demonstrate that the ESKD diminishes the activity of AMPKα as well as the expression levels of other proteins and transcription factors regulating mitochondrial biogenesis but increases anti-oxidant SOD2 expression as a defense mechanism against mitochondrial ROS, which is probably elevated due to the disturbed oxidative hemostasis during ESKD. Our data also suggest abnormal mitochondrial dynamics in disease as evidenced by low expression levels the fission (DRP1) and fusion (OPA1) proteins. Earlier studies have also attributed mitochondrial dysfunction to disease etiologies in the context of kidney diseases (42, 66) but the metabolic genotype of the cells (e.g., bioenergetic parameters, shift between respiration and glycolysis, etc.) have been characterized in more detail in our study. Overall, our protocol and data provide an evidence for kidney mitochondrial metabolism may be related to the bioenergetic profiles of blood cells.

## Conclusion

We described a methodology for assessing mitochondrial respiration phenotypes by incorporating instrumentation and standardization of extracellular flux protocols. Our strategy exhibited promising performance in terms of measuring the function of mitochondria in patients (30, 67) and allowed us to compare mitochondrial bioenergetics in health and disease. Accounting for differences in bioenergetic capacities of ESKD patients and healthy controls is important because such an information provides more insight into why certain people are at high risk of renal disease development and progression. This will improve our understanding of the disease mechanism and help us to identify new diagnostic targets and develop safer therapies for ESKD patients under dialysis treatment.

## Data Availability Statement

The original contributions presented in the study are included in the article/supplementary material. Further inquiries can be directed to the corresponding author.

## Ethics Statement

The studies involving human participants were reviewed and approved by Institutional Review Board of Rush University Medical Center. The patients/participants provided their written informed consent to participate in this study.

## Author Contributions

MA, SD, LT, BS and HW conceived and designed the studies. MA, SD and BS performed experiments. MA and SD analyzed data and drafted the manuscript. HW revised the manuscript. All authors provided comments on the manuscript and approved the submitted version. All authors contributed to the article and approved the submitted version.

## Conflict of Interest

The authors declare that the research was conducted in the absence of any commercial or financial relationships that could be construed as a potential conflict of interest.
